# Effects of chironomid larvae density and mosquito biocide on methane and carbon dioxide dynamics in freshwater sediments

**DOI:** 10.1371/journal.pone.0301913

**Published:** 2024-05-24

**Authors:** Caroline Ganglo, Alessandro Manfrin, Clara Mendoza-Lera, Andreas Lorke

**Affiliations:** iES Landau, Institute for Environmental Sciences, RPTU Kaiserslautern-Landau, Landau, Germany; University of Siena, ITALY

## Abstract

Small lentic water bodies are important emitters of methane (CH_4_) and carbon dioxide (CO_2_), but the processes regulating their dynamics and susceptibility to human-induced stressors are not fully understood. Bioturbation by chironomid larvae has been proposed as a potentially important factor controlling the dynamics of both gases in aquatic sediments. Chironomid abundance can be affected by the application of biocides for mosquito control, such as Bti (*Bacillus thuringiensis* var. *israelensis*). Previous research has attributed increases in CH_4_ and CO_2_ emissions after Bti application to reduced bioturbation by chironomids. In this study, we separately tested the effect of chironomid bioturbation and Bti addition on CH_4_ production and emission from natural sediments. In a set of 15 microcosms, we compared CH_4_ and CO_2_ emission and production rates with high and low densities of chironomid larvae at the bioturbating stage, and standard and five times (5x) standard Bti dose, with control sediments that contained neither chironomid larvae nor Bti. Regardless of larvae density, chironomid larvae did not affect CH_4_ nor CO_2_ emission and production of the sediment, although both rates were more variable in the treatments with organisms. 5xBti dosage, however, led to a more than three-fold increase in CH_4_ and CO_2_ production rates, likely stimulated by bioavailable dissolved carbon in the Bti excipient and priming effects. Our results suggest weak effects of bioturbating chironomid larvae on the CH_4_ and CO_2_ dynamics in aquatic ecosystems. Furthermore, our results point out towards potential functional implications of Bti for carbon cycling beyond those mediated by changes in the macroinvertebrate community.

## 1. Introduction

Freshwater ecosystems are the largest natural source of methane (CH_4_) [1] and carbon dioxide (CO_2_) [2] and small, shallow aquatic systems are hotspots of carbon cycling [3], by contributing about 41% and 15% to the global diffusive CH_4_ and CO_2_ emissions from lentic water bodies [2]. CH_4_ is mostly produced in anoxic sediments as a function of the availability of labile carbon and alternative terminal electron acceptors, and temperature [4–6]. Under aerobic conditions, CH_4_ can be oxidized to CO_2_ [7]. CO_2_ is mainly produced by aerobic respiration [8]. Tube-dwelling macroinvertebrates, such as the most widely distributed Chironomidae (hereafter referred to as chironomid larvae), rework and ventilate sediments [9]. These bioturbating activities promote aerobic processes in the upper sediment [10–12], and potentially modulate CH_4_ and CO_2_ dynamics [9]. Burrow construction and ventilation is limited to the latest larval stage of chironomids (third to fourth instar) [12].

Experimental evidence for the effects of chironomid larvae density and activity on CH_4_ and CO_2_ production and emission across the sediment-water interface, however, is limited and findings are contrasting. Like other benthic macroinvertebrates and benthivorous fish, chironomid larvae may affect the dynamics of CO_2_ and CH_4_ through trophic and non-trophic interactions [13]. Potential trophic interactions include the feeding on organic matter [14] and microbial community (including CH_4_ oxidizing bacteria) [15, 16]. Non-trophic effects include enhanced transport of nutrients and oxygen (O_2_) into the sediment, increasing the volume of oxic sediment, and aerobic respiration [13, 17–19]. On the one hand, laboratory incubations of paddy soils showed that chironomid larvae had no effect on CH_4_ emission across the sediment-water interface, neither by diffusion nor by ebullition [20]. On the other hand, non-linear responses in both CH_4_ and CO_2_ emissions were found in experiments in which bioturbation was mimicked by periodic mechanical disturbances of the sediment [17]. Higher emissions from disturbed sediment were predominantly linked to the initiation of gas bubble release (CH_4_ ebullition).

The widespread application of the biocide *Bacillus thuringiensis* var. *israelensis* (Bti) for mosquito control [21–24] has been reported to reduce the larval density of non-target organisms [25–27], including chironomid larvae [28]. The reduction of chironomid larvae density due to Bti was proposed as a possible reason for higher CH_4_ emissions from mesocosms treated with Bti [29]. The authors proposed that the decrease in chironomid larvae density may affect both CH_4_ and CO_2_ dynamics via alterations in bioturbation activity. Furthermore, it has been suggested that the excipient of Bti contains bioavailable dissolved organic carbon [30] that could potentially boost aerobic respiration and methanogenesis for a given limited time [5, 31, 32]. Experimental evidence for these conjectures from former studies, however, is lacking, as the specific effect pathway by which Bti addition has affected the CH_4_ emissions in the mesocosm experiments could not be disentangled.

To analyze the relative importance of changes in chironomid larvae density and Bti addition, we separately tested the effect of both on CH_4_ and CO_2_ dynamics in freshwater sediments using model systems (i.e. laboratory microcosms). We hypothesized that increasing chironomid larvae density would result in decreasing CH_4_ production and emission due to increased bioturbating activity that reduces anoxic sediment volume (increasing CO_2_ emissions from aerobic respiration). We further hypothesized that the addition of Bti in absence of chironomid larvae would stimulate the production and emission of CH_4_ and CO_2_ due to addition of labile organic carbon.

## 2 Material and methods

### 2.1 Experimental design

**Fig 1:**
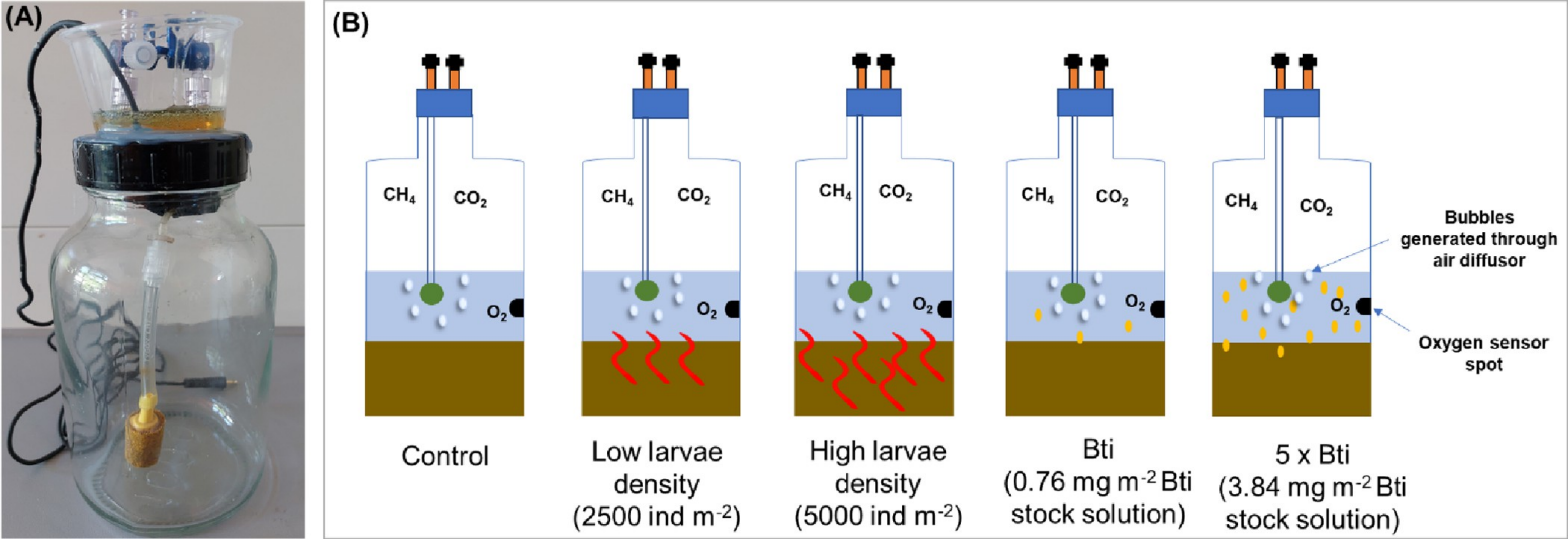
Experimental microcosms (A), and experimental design (B) with four treatments and control: Control, Low, and High chironomid larvae density, Bti, and 5 x Bti. Each treatment and the control had three replicates.

We conducted the experiment in gas-tight microcosms (1 L glass bottles, height 17.8 cm, radius 4.5 cm, area 63.8 cm^2^) containing 0.19 L of sediment (3 cm height), 0.2 L of overlying water (3 cm height), and 0.61 L of headspace (**Fig 1A**). We sampled the headspace gas for measuring CH_4_ and CO_2_ concentrations through a connector in the lid and measured dissolved O_2_ concentration in the water through contactless sensors (PyroScience GmbH, Germany) at 2 cm above the sediment surface. The relatively large headspace volume provided sufficient oxygen for maintaining the concentration of dissolved oxygen in the water well above 30% saturation, which has been reported as a threshold for alterations of the bioturbating activity of chironomid larvae [33, 34]. To ensure gas equilibration between the water and the headspace, we continuously aerated the water with headspace air using an internal air pump and a submerged bubble diffusor (**Fig 1**). The gas recirculation rate (150 mL h^-1^) generated gentle flows in the water, without causing sediment resuspension.

We collected sediment and water from a pond at the Eußerthal Ecosystem Research Station (49°15’16" N, 7°57’42" E, facility of the University of Kaiserslautern-Landau, Germany, no permits were required). We sieved the sediment (mesh size 0.2 mm) to remove debris and large organisms and acclimated it to the experimental conditions (25°C ± 2°C, in darkness) for 10 days.

The experimental design consisted of four treatments and one control, each replicated three times (**Fig 1B**): i) low, and ii) high chironomid larvae density, iii) standard Bti concentration (no chironomid larvae); iv) 5 X standard Bti concentration (no chironomid larvae). The control consisted of sediment and overlying water without chironomid larvae or Bti. The treatments with chironomid larvae contained 16 (Low larvae density) and 32 (High larvae density) *Chironomus riparius* third to fourth instar larvae (**Fig 1B,** the experiment with invertebrates required no special permission). Third to fourth instar larvae (10–15 mm body length) are known to bioturbate before emerging as adults [20, 37]. These larvae densities used in our experiments correspond to 2500 and 5000 individuals per m^2^, which is in the mid-range of densities found in shallow lake sediments (70–110,00 individuals per m^2^, [35]). The larvae were cultured following OECD (Organisation for Economic Co-operation and Development) guidelines [36] in three aquaria (4 L, height 8 cm, surface area 588 cm^2^) containing 200 mL of a mixture of sediment consisting of peat, kaolin clay, and sand [36], purchased from a local garden center, and one liter of Borgmann medium (174 mg CaCl_2_·2H_2_O, 85.5 mg NaHCO_3_, 61.5 mg MgSO_4_·7H_2_0, 1.03 mg NaBr and 3.8 mg KCl in 1 L of deionized water). Once per week, we replaced the culture medium, and fed the chironomid larvae with 23.52 g of fish food (Tetramin flakes).

The Bti treatments consisted of one at the standard field application rate (2.9 x 10^9^ International Toxic Units (ITU; [37, 38]) and another at five times that rate (hereafter referred to as Bti and 5 x Bti, respectively, **Fig 1**). To distinguish dose effects from the actual action of Bti on biogeochemistry, we additionally selected a higher dose than the recommended (standard) application rate. To each treatment, we added 1 mL of a Bti stock solution containing 0.76 mg mL^-1^ and 3.84 mg mL^-1^ of VectoBac WDG (2400 ITU mg^-1^) (Valent BioSciences Corporation, Illinois, USA) (**Fig 1**). We estimated the amount of dissolved organic carbon (DOC) contained in the Bti stock solutions by catalytic combustion, after acidification with hydrochloric acid (HCl) to remove inorganic carbon, following DIN EN ISO/IEC 17025:2018. We added 0.18 ± 0.02 mg DOC (15 ± 1.6 μmol carbon) to the standard Bti treatment and 0.84 ± 0.12 mg DOC (70 ± 10 μmol carbon, all values as mean ± standard deviation) to the 5 x Bti treatment. These additions correspond to increases in surface water DOC concentrations of 0.91 ± 0.10 mg L^-1^ and 4.24 ± 0.61 mg L^-1^. We determined specific ultraviolet absorbance (SUVA_254_) of filtered (pore size 0.45 μm, Altmann Analytik PA4525-100) Bti solution using an UV–VIS scanning spectrophotometer (T3,15A/H, 232B105 Analytik Jena AG Germany) in a 1 cm cuvette and at a wavelength of 254 nm. We determined the SUVA_254_ (3.18 ± 0.16 L mg^-1^ m^-1^) as the ratio of the measured absorbance (0.29 ± 0.05 cm^-1^) and the DOC concentration (9.15 ± 1.09 mg L^-1^) in the sample multiplied by 100.

### 2.2 CO_2_ and CH_4_ emission and net production and oxygen consumption

Prior to the start of the experiment, we filled all microcosms (plus three additional ones for initial concentration determination, see below) with sediment and sterile-filtered pond water and let them settle for 72 hours open to the atmosphere. After 72 hours, we added the chironomid larvae and Bti to the corresponding microcosms (**Fig 1B**) and closed them gas-tight. We used the three additional microcosms to determine the total initial amount of CH_4_ and CO_2_ in the pore water and in the surface water of the microcosms, by measuring the headspace concentrations after vigorous shaking (to equilibrate the pore and surface water with the headspace). These microcosms were subsequently discarded. At 24 h, 72 h, and 120 h after the start of the experiment, we measured dissolved O_2_ concentration, and the CO_2_ and CH_4_ mixing ratios in the headspace to determine O_2_ consumption, and CO_2_ and CH_4_ emission rates. We ended the experiment when we detected the first emerged adult chironomid after 120 h. After the last headspace sampling at 120 h, we vigorously shook all microcosms to ensure full equilibration between pore water, surface water, and the headspace, and collected gas samples from the headspace to determine the total amount of CH_4_ and CO_2_ in the microcosms (dissolved and gaseous). We used the difference between the total amount at the end and at the beginning of the experiment to determine the net production rates of CO_2_ and CH_4_, which also account for the gas that has accumulated in the pore water during the experiment.

At each sampling (24 h, 72 h, and 120 h after the start of the experiment), we collected 100 μL of headspace gas from each microcosm using a gastight syringe (Hamilton, USA). The mixing ratios (ppmv) of CO_2_ and CH_4_ were measured by injecting the samples into a gas analyzer (Ultra-portable Greenhouse Gas Analyzer; UGGA, Los Gatos Research Inc., Mountain View, CA, USA) in closed-loop operation [39]. By assuming full equilibration between the headspace and the overlying water, we determined the amount of CH_4_ and CO_2_ (μmol) in the headspace and overlying water at each sampling time. We estimated gas-specific Henry coefficients at incubation temperature using common parametrizations [40]. We then calculated the emission rates of CH_4_ and CO_2_ as the difference in the amount between two subsequent samplings divided by the elapsed time. The emission rates account for the fluxes across the sediment-water interface and potential oxidation of CH_4_ and CO_2_ production by respiration in the surface water.

After each headspace sampling, we measured dissolved O_2_ concentration in the overlying water. We calculated the amount of O_2_ (μmol) as the sum of O_2_ gas in the headspace and dissolved O_2_ in the overlying water by assuming equilibrium between the water and the headspace. We calculated O_2_ consumption rates (i.e., respiration) from the difference between two subsequent samplings divided by the elapsed time.

We estimated the total net production rates of CH_4_ and CO_2_ as the difference between the mean total amount of CH_4_ and CO_2_ at the beginning and at the end of the experiment (after shaking), divided by the total experimental time (120 h). The net CH_4_ and CO_2_ production rates account for the emissions and for gas that accumulated in the sediment during the incubation period.

As a reference for other studies, the estimated production and emission rates were additionally normalized by sediment surface area (63.8 × 10^−3^ m^2^) and sediment dry weight measured for three control vessels at the end of the experiment (72.07 ± 4.51 g), respectively. These values are provided in **S1 Table**.

### 2.3 Statistical analysis

We used a linear mixed effect model implemented in the lme function from the nlme package (version 3.1–164) [41] for R [42] to test for differences in amount, emission, and net production rates of CH_4_, CO_2_, the ratios of emission to net production rate, and O_2_ consumption rate among treatments (control, low and high chironomid larvae density, Bti and 5x Bti). For each variable, we considered treatment as a fixed factor, and replicated microcosms and sampling time as random factors (except for the net CH_4_ and CO_2_ production rates, which were not replicated in time). We assessed model residuals using quantile-quantile plots and plots of residuals versus fitted values. When needed, we log-transformed dependent variables to meet assumptions of normality and homogeneity. For significant effects, we applied a post-hoc contrast analysis using the lsmeans function from the lsmeans package (version 3.1–164) [41] for R. To decrease the false discovery rate due to multiple testing, we adjusted p-values using the Benjamini-Hochberg correction [43].

## 3 Results

**Fig 2:**
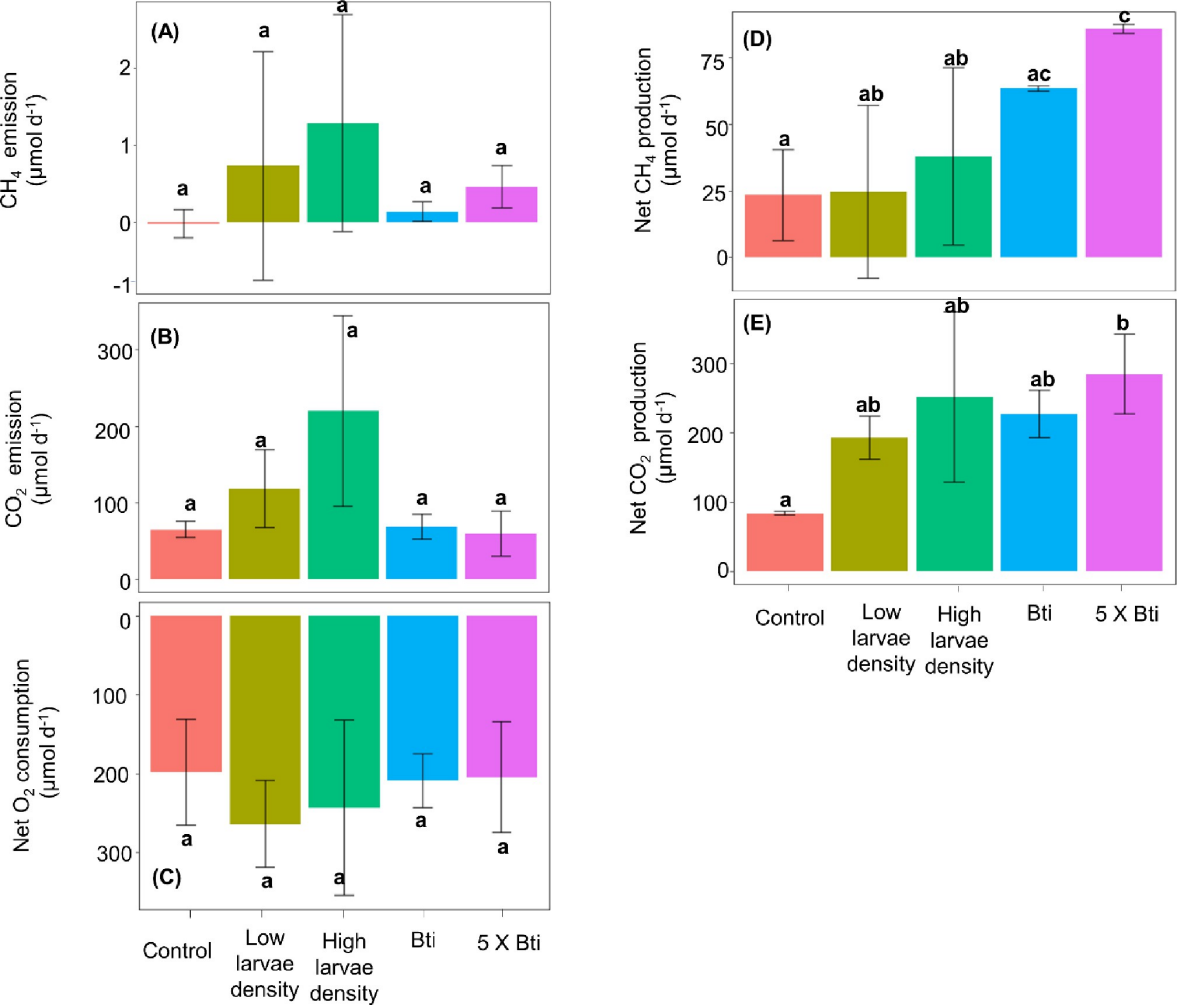
Mean ± Standard Deviation emission rates (*n* = 3) of CH_4_ (**A**), CO_2_ (**B**), and O_2_ consumption (**C**), and net production rates (*n* = 3) of CH_4_ (**D**) and CO_2_ (**E)** for each treatment (Control, Low larvae density, High larvae density, Bti, and 5 x Bti). Significant differences (*p* ≤ 0.05) between treatments are indicated by different letters.

Throughout the experiment, the surface water remained well oxygenated with a mean concentration of dissolved O_2_ in the overlying water for all treatment of 7.0 ± 0.7 mg L^-1^ (corresponding to 81 ± 8% O_2_ saturation, with a minimum value of 63%) at the last sampling (**S1 Fig**). Overall, we observed CH_4_ and CO_2_ production, and O_2_ consumption in all treatments (**Fig 2**). For chironomid larvae density treatments, the emission of both CO_2_ and CH_4_ were highly variable compared to the control and tended to increase with chironomid larvae density, yet no significant differences were found (**Fig 2A and 2B; Table 1 and S2 Table**). O_2_ consumption was not affected by chironomid larvae density (**Fig 2C**). Alike the emissions, net production rates of CO_2_ and CH_4_, which additionally consider gas that has accumulated in the sediment, were comparable between the control and treatments with low and high chironomid larvae densities, which were more variable than the Bti and 5 x Bti treatments (**Fig 2D and 2E; Table 1; S2 and S3 Tables**).

**Table 1:** Linear mixed effect model for the effects of treatment on CH_4_, CO_2_ emission and net production, and O_2_ consumption.

Variables	Factors	numDF	denDF	*F*	*p*
CH_4_ emission (μmol d^-1^)	Treatment	4	10	0.93	0.48
**CO**_**2**_ **emission** (μmol d^-1^)	**Treatment**	**4**	**10**	**3.55**	**0.04**
O_2_ consumption (μmol d^-1^)	Treatment	4	10	0.47	0.75
**Net CH**_**4**_ **production** (μmol d^-1^)	**Treatment**	**4**	**10**	**4.47**	**0.02**
**Net CO**_**2**_ **production** (μmol d^-1^)	**Treatment**	**4**	**10**	**4.35**	**0.02**
Ratio CH_4_ emission/ net production	Treatment	4	10	0.69	0.61
**Ratio CO**_**2**_ **emission/ net production**	**Treatment**	**4**	**10**	**13.48**	**5x10** ^**-4**^

Significant differences at *p* < 0.05 are indicated in bold letters (*F*-statistics, numDF: numerator degrees of freedom; denDF: denominator degrees of freedom). See the complete results of the statistical analysis in **S1–S3 Tables.**

The addition of Bti, did neither affect CO_2_ and CH_4_ emission, nor O_2_ consumption compared to the control (**Fig 2A–2C; Table 1; S2 Table**). However, Bti promoted significantly higher net production of CH_4_ and CO_2_ at the 5x dose, whereas no effect was observed at the standard dose (**Fig 2D and 2E and S2 and S3 Tables**). Compared to the lack of effects of chironomid larvae density, Bti had an effect at the 5 x dose on net CH_4_ production, but not on CO_2_ production (**Fig 2D and 2E; Table 1; S2 and S3 Tables**).

**Fig 3:**
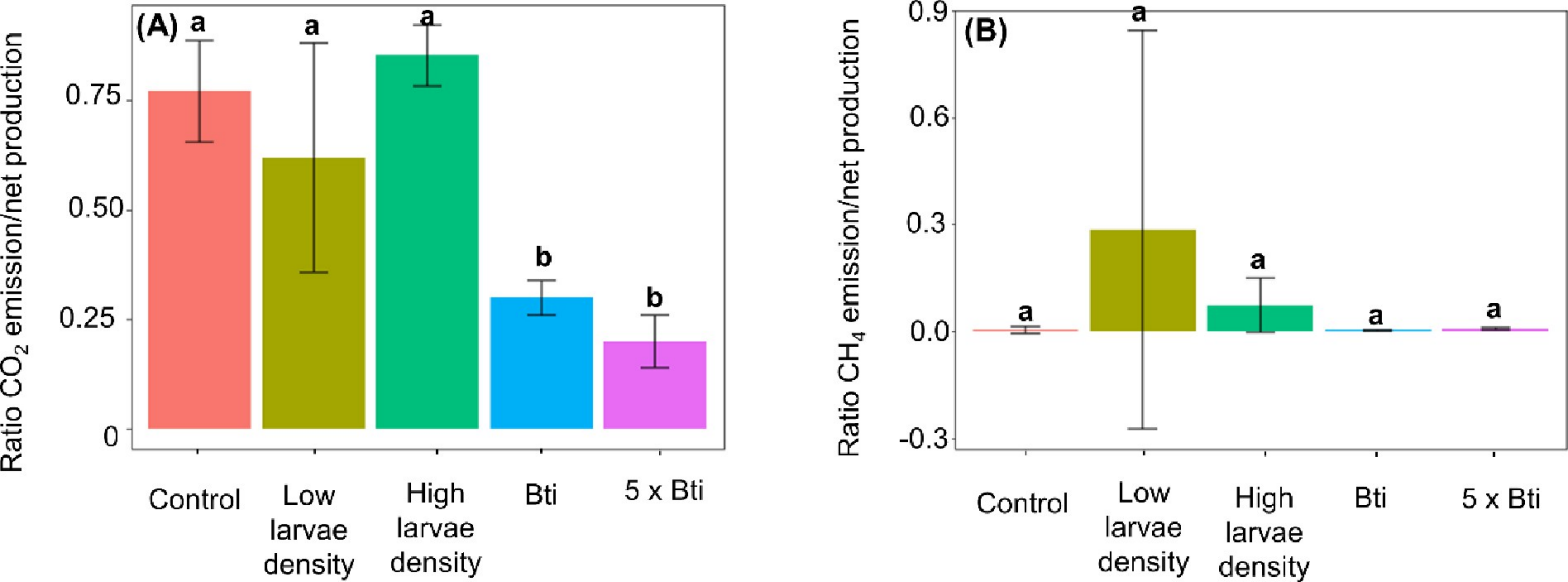
Mean ± Standard Deviation ratios of emission-to-net production rates (*n* = 3) of CO_2_ (**A**) and CH_4_ (**B**) for each treatment (Control, Low larvae density, High larvae density, Bti, and 5 x Bti). Significant differences (*p* ≤ 0.05) between treatments are indicated by different letters.

The ratio of CO_2_ emission to net production for both chironomid larvae densities was similar to that observed in the control (**Fig 3A; Table 1; S2 and S3 Tables**), indicating that most of the CO_2_ produced during the experiment was emitted to the overlying water and headspace. In the Bti treatments, the ratio of CO_2_ emission and production rates was significantly lower than in the control, and the net production rates exceeded the emission by a factor of three (**Fig 3A**). This smaller ratio was mostly caused by the higher net production, whereas the emission rates were more similar. Overall, the ratio of CH_4_ emission to net production was comparable among treatments (**Fig 3B; Table 1; S2 Table**). For CH_4_, the ratio of emission to net production was smaller than 1% for both Bti treatments and the control, indicating that most of the produced CH_4_ had accumulated in the sediment (**Fig 3B**). For the treatments with chironomid larvae, the ratio was highly variable and reached almost 10% in one replicate (**Fig 3A; 3B; S3 Table**). The tendency towards higher ratios of CH_4_ emission to net production rates in these treatments in comparison to the control was mostly caused by higher emission rates, whereas the production rates were more similar (**Fig 2A and 2D**).

## 4 Discussion

### 4.1 Effects of bioturbation on CH_4_ and CO_2_ dynamics in aquatic sediments

We hypothesized that higher density of chironomid larvae would result in lower net production and emission of CH_4_ due to their bioturbating activity (pumping of oxygenated water into the sediment). As bioturbation would reduce the volume of anoxic sediment and increase the volume of oxic sediment with aerobic metabolism, we further expected higher net production and emission rates of CO_2_. Contrary to our hypotheses, we found no significant effect of the presence of chironomid larvae in high or low density on CH_4_ or CO_2_ emission and production rates in comparison to sediment without larvae (control). Former studies simulated bioturbation by mechanical disturbances of the sediment surface and found an enhancement of CH_4_ transport from porewater to overlying water associated with ebullitive emission, but not for diffusive fluxes [17]. Contrary to these findings, our experiments with actual macroinvertebrate activity indicate that bioturbation might not affect the total emissions (diffusive and ebullitive), as also reported by another study with chironomid larvae [20]. However, we found high variability among replicates for the two treatments involving chironomid larvae and a tendency, though not significant, towards higher CH_4_ and CO_2_ emission rates compared to the control. The variability may have challenged our capacity to detect clear treatment effects, which would become significant only for a larger number of replicates. High variability in sediment respiration rates in the presence of chironomid larvae has also been reported from previous experiments in microcosms of comparable size as in our study [11]. The variability can potentially be related to intraspecific variations in chironomid larvae activity, as the duration and frequency of burrow ventilation can vary strongly among individual organisms [10].

The variability among replicates was highest for CH_4_ emissions and for the ratio of emission to net production of CH_4_. The more pronounced variability in CH_4_ as compared to CO_2_ can be explained by the spatial separation of aerobic respiration and CH_4_ production in the microcosms. As CO_2_ is predominantly produced by aerobic respiration in the oxygenated surface sediment, it was more rapidly transported to the overlying water. CH_4_ production took place in deeper sediment layers, where it accumulated during the experiment. In addition, also the respiration of the chironomid larvae contributed directly to net CO_2_ production and emission rates, as well as to O_2_ consumption. Chironomid larvae respire between 2.9 and 6.5 μmol O_2_ per day [9]. Assuming an average of 5 μmol per day and individual, the net O_2_ consumption by chironomid larvae was 80 and 180 μmol O_2_ per day for our low and high chironomid larvae densities, respectively. For a unity respiratory quotient, these rates fall in the range of the observed increase in CO_2_ emission in both treatments involving chironomid larvae compared to the control, suggesting that the tendency towards higher CO_2_ emission and production was predominantly caused by chironomid larvae respiration, rather than by a stimulation of microbial metabolism in the sediment by bioturbation.

The tendency of higher ratios of CH_4_ emission to net production rates in the presence of chironomid larvae resulted mostly from higher emission rates during the experiment and reduced accumulation of CH_4_ in the sediment. Although our experimental approach did not allow to distinguish ebullitive and diffusive fluxes and ebullition was not observed visually during the sampling, the release of a single gas bubble may have resulted in a large change in the headspace CH_4_ concentrations of the microcosms and the estimated emission rates. Both burrow construction by chironomid larvae and gas bubble formation in the sediment result in spatial heterogeneity of the sediment pore structure at the centimeter scale [44, 45], i.e. at a scale comparable to the size of our microcosms. Therefore, the relatively small size of the microcosms, in relation to this heterogeneity, may have additionally contributed to the high variability among replicates due to insufficient spatial integration. Overall, the potential of bioturbation by chironomid larvae to affect emission rates seems to be strongly modulated by interacting physical and biological factors.

### 4.2 Effects of Bti on CH_4_ and CO_2_ dynamics in aquatic sediment

To the best of our knowledge, our study provides the first evidence of functional implications of the mosquito biocide Bti in biogeochemical cycling in sediments from a freshwater aquatic ecosystem. Previous research was limited to the effect on the structure of planktonic communities [46], or could not distinguish Bti effects from those induced by changes in the macroinvertebrate density [29, 30]. We hypothesized that Bti excipients could represent a pulse of labile dissolved organic carbon (DOC), temporarily promoting aerobic (CO_2_ dynamics) and anerobic (CH_4_ dynamics) carbon processing. Our results showed that the addition of Bti at high dose (5x standard dose) increased net CO_2_ and CH_4_ production, indicating that both aerobic and anaerobic carbon metabolism might have been boosted by labile carbon availability upon Bti addition. The increase in net CO_2_ production in Bti treatments was comparable to that due to chironomid larvae activity. In comparison to the treatments with chironomid larvae and to the control, a larger fraction of the produced CO_2_ was stored in the sediment, i.e., the emission to net production ratio was reduced, indicating that at least parts of the source of the additional CO_2_ was in deeper layers of the sediment.

The amount of carbon added with Bti was about one order of magnitude smaller than the difference in C-CO_2_ (792 μmol) and C-CH_4_ (321 μmol) produced in the 5xBti relative to the control. Consequently, the higher net CO_2_ and CH_4_ production rates in Bti treatments cannot be explained solely by the decomposition of the DOC added with Bti, even if it was completely metabolized during the five-day experimental period. The discrepancy could be explained by a stimulation of aerobic and anaerobic degradation of sediment organic carbon that was not available without the presence of Bti. This could be either through Bti-induced changes in the composition of the microbial community [47] or through priming effects [48] of the dissolved organic carbon in Bti excipients.

## 5 Conclusions

Results from a recent mesocosm study showed increased CH_4_ emissions upon treatment with the biocide Bti [29], which is widely applied to lentic freshwater bodies for mosquito control. In this previous study, the authors hypothesized that the increased emissions, which persisted for several months after Bti addition, were caused by a reduction in the density of chironomid larvae by Bti. In this study, we separately tested for the effect of chironomid larvae bioturbation and Bti addition on CH_4_ and CO_2_ production and emission from natural sediments in microcosm experiments. Our results suggest complex interactions between bioturbating chironomid larvae and CH_4_ and CO_2_ dynamics in aquatic sediments that need further research. Therefore, we cannot confirm this conjecture from the former observations. On the other hand, however, our results reveal a potential direct effect of Bti and its recipient on microbial communities and their activity resulting in higher CH_4_ emissions from treated water bodies. Consequently, our study adds to the growing, yet very limited, evidence that chemical and microbial stressors can have adverse effects on carbon dynamics and greenhouse gas emissions in affected aquatic ecosystems [29, 49–51]. The biogeochemical implications of these stressors should receive more attention in assessments of their environmental impacts.

## Supporting information

S1 Fig Mean ± Standard Deviation of amount (μmol) of (**A**) CH_4_, (**B**) CO_2_, and (**C**) O_2_ emitted for Control, Low and High Density, Bti and 5 x Bti at 24 h, 72 h, and 120 h during the incubation period (*n* = 3), and the total final amount produced over the experiment (including dissolved and gaseous component in the sediment porewater) of (**D**) CH_4_ and (**E**) CO_2_ (*n* = 3). Different letters above bar indicate significant differences within treatment at the same timepoint (*p* ≤ 0.05).
(TIF)

S1 Table Mean values and standard deviation of gas emission and production per sediment srface area (μmol d^-1^ m^-2^) and per dry weight (μmol d^-1^ g^-1^). (PDF)

S2 Table Linear mixed effect model selection using likelihood ratio tests (LRT) against reduced models. Treatment effects were tested for CH_4_ and CO_2_ emission and net production, and O_2_ consumption. Statistically significant differences are marked in bold (*p* ≤ 0.05).
(PDF)

S3 Table Post-hoc pairwise comparisons of CH_4_ and CO_2_ emission and net production, and O_2_ consumption rates. The estimates represent the mean difference between pairwise factors and *t*-ratio is the ratio of the estimate and the standard error. Statistically significant differences are marked in bold (*p* ≤ 0.05).
(PDF)
