# Supplementary figures and images for: Effects of chironomid larvae density and mosquito biocide on methane and carbon dioxide dynamics in freshwater sediments
Source: PLoS One. 2024 May 24;19(5):e0301913. doi: 10.1371/journal.pone.0301913 (PMC11125464; doi:10.1371/journal.pone.0301913)

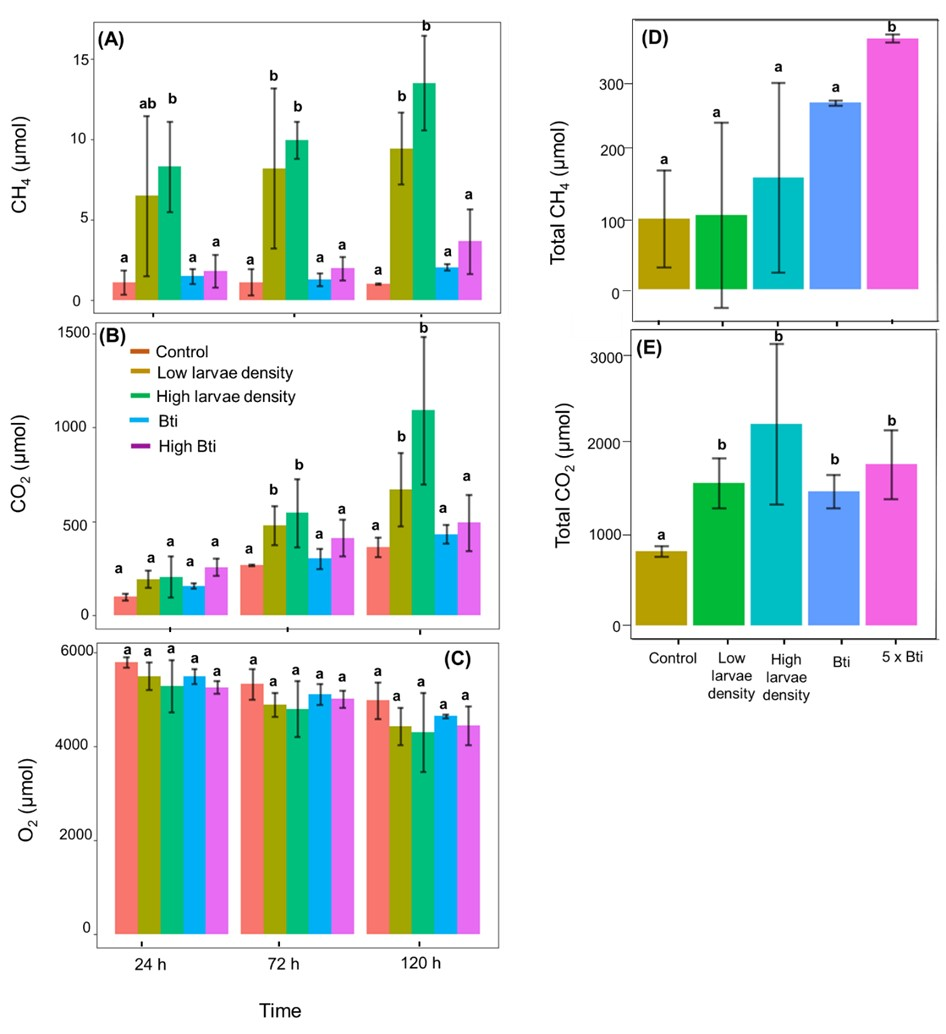

Supplement: S1 Fig — Mean ± Standard Deviation of amount (μmol) of (A) CH4, (B) CO2, and (C) O2 emitted for Control, Low and High Density, Bti and 5 x Bti at 24 h, 72 h, and 120 h during the incubation period (n = 3), and the total final amount produced over the experiment (including dissolved and gaseous component in the sediment porewater) of (D) CH4 and (E) CO2 (n = 3). Different letters above bar indicate significant differences within treatment at the same timepoint (p ≤ 0.05). (TIF) [file pone.0301913.s001.tif]
