# Supplementary material for: Effects of chironomid larvae density and mosquito biocide on methane and carbon dioxide dynamics in freshwater sediments
Source: PLoS One. 2024 May 24;19(5):e0301913. doi: 10.1371/journal.pone.0301913 (PMC11125464; doi:10.1371/journal.pone.0301913)
Supplement: S1 Table — (PDF) [file pone.0301913.s002.pdf]

S1 Table: Mean values and standard deviation of gas emission and net production rates normalized by sediment surface area (µmol d^{-1} m^{-2}) and by sediment dry weight (µmol d^{-1} g^{-1})

Variables	Treatment				
	Control	Low larvae density	High larvae density	Bti	5xBti
**Emission rates**					
CH_4 (µmol d^{-1} m^{-2})	-3.29 ± 29.06	202.01 ± 220.76	114.77 ± 232.76	21.85 ± 19.60	72.00 ± 43.27
CH_4 (µmol d^{-1} g^{-1})	-0.0003 ± 0.003	0.02 ± 0.02	0.01 ± 0.02	0.002 ± 0.002	0.006 ± 0.004
CO_2 (µmol d^{-1} m^{-2})	10113.70 ± 1726.85	34428.80 ± 19539.60	18513.20 ± 8048.99	10681.80 ± 2530.91	9223.47 ± 4622.51
CO_2 (µmol d^{-1} g^{-1})	0.89 ± 0.15	3.05 ± 1.73	1.64 ± 0.71	0.94 ± 0.22	0.82 ± 0.41
O_2 (µmol d^{-1} m^{-2})	-31032 ± 10517	-38163 ± 17424.30	-41413 ± 8616.20	-32811 ± 5406.34	-32040 ± 11028.50
O_2 (µmol d^{-1} g^{-1})	-2.75 ±	-3.38 ±	-3.67 ±	-2.90 ±	-2.84 ±

Variables	Treatment				
	Control	Low larvae density	High larvae density	Bti	5xBti
	0.93	1.54	0.76	0.48	0.98
**Net production rates**					
CH_4 (µmol d^{-1} m^{-2})	3663.37 ± 2668.06	5919.08 ± 5202.16	3845.45 ± 5062.39	9928.06 ± 150.527	13426.60 ± 265.597
CH_4 (µmol d^{-1} g^{-1})	0.32 ± 0.24	0.52 ± 0.46	0.34 ± 0.45	0.88 ± 0.01	1.19 ± 0.02
CO_2 (µmol d^{-1} m^{-2})	13071.90 ± 380.98	39420.60 ± 19245	30250.50 ± 4862.06	35567.50 ± 5391	44565 ± 8999.71
CO_2 (µmol d^{-1} g^{-1})	1.16 ± 0.03	3.49 ± 1.70	2.68 ± 0.43	3.15 ± 0.48	3.94 ± 0.80
