# Supplementary material for: Effects of chironomid larvae density and mosquito biocide on methane and carbon dioxide dynamics in freshwater sediments
Source: PLoS One. 2024 May 24;19(5):e0301913. doi: 10.1371/journal.pone.0301913 (PMC11125464; doi:10.1371/journal.pone.0301913)
Supplement: S2 Table — Treatment effects were tested for CH4 and CO2 emission and net production, and O2 consumption. Statistically significant differences are marked in bold (p ≤ 0.05). (PDF) [file pone.0301913.s003.pdf]

S2 Table: Linear mixed effect model selection using likelihood ratio tests (LRT) against reduced models. Treatment effect on CH_4 and CO_2 emission and net production, and O_2 consumption. Statistically significant differences are marked in bold (*p* ≤ 0.05).

Variables	R^2 marginal	Factors	*F*-value ($_{numDF, denDF}$)	*p*-value
	Likelihood ratio test (LRT) against reduced model		LRT	
CH_4 emission (µmol d^{-1})	0.21	Treatment	7.72	0.10
			$F_{4,10}$=0.93	0.48
**CO_2 emission** (µmol d^{-1})	**0.50**	**Treatment**	**49.31**	**<.0001**
			**$F_{4,10}$=3.55**	**0.04**
O_2 consumption (µmol d^{-1})	0.11	Treatment	7.72	0.10
			$F_{4,10}$=0.47	0.75
**Net CH_4 production** (µmol d^{-1})	**0.56**	**Treatment**	**42.97**	**<.0001**

Variables	R^2 marginal	Factors	*F*-value ($_{numDF, denDF}$)	*p*-value
			**$F_{4,10}$=4.47**	**0.02**
**Net CO_2 production** (µmol d^{-1})	**0.55**	**Treatment**	**51.25**	**<.0001**
			**$F_{4,10}$=4.35**	**0.02**
Ratio CH_4 emission/ net production	0.16	Treatment	3.71	0.44
			$F_{4,10}$=0.69	0.61
**Ratio CO_2 emission/ net production**	**0.79**	**Treatment**	**13.87**	**0.007**
			**$F_{4,10}$=13.48**	**5e-04**
