# Supplementary material for: Effects of chironomid larvae density and mosquito biocide on methane and carbon dioxide dynamics in freshwater sediments
Source: PLoS One. 2024 May 24;19(5):e0301913. doi: 10.1371/journal.pone.0301913 (PMC11125464; doi:10.1371/journal.pone.0301913)
Supplement: S3 Table — The estimates represent the mean difference between pairwise factors and t-ratio is the ratio of the estimate and the standard error. Statistically significant differences are marked in bold (p ≤ 0.05). (PDF) [file pone.0301913.s004.pdf]

S3 Table: Post-hoc pairwise comparisons of CH_4 and CO_2 emission and net production, and O_2 consumption. The estimates represent the mean difference between pairwise factors and t-ratio is the ratio of the estimate and the standard error. Statistically significant differences are marked in bold ($p \leq 0.05$).

Variables	Contrast	Pairwise structure	Estimate	*t*-value	*p*	*p*-adjusted
CO_2 emission (µmol d^{-1})	Treatment	Low larvae density - Control	53.59	1.053	0.4992	0.8256
		High larvae density - Control	155.13	3.048	0.0463	0.0729
		Bti - Control	3.62	0.071	0.9446	1
		5xBti - Control	-5.68	-0.112	0.9446	1
		High larvae density - Low larvae density	-101.54	1.995	0.185	0.3338
		Bti - Low larvae density	-49.96	-0.982	0.4992	0.8576
		5 x Bti – Low larvae density	-59.27	-1.164	0.4992	0.7704
		Bti – High larvae density	-151.51	-2.977	0.0463	0.0813
		5 x Bti – High larvae density	-160.81	-3.159	0.0463	0.0613
		5 x Bti - Bti	-9.3	-0.183	0.9446	0.9997
Net CH_4 production (µmol d^{-1})	Treatment	Low larvae density - Control	1.16	0.064	0.9499	1

Variables	Contrast	Pairwise structure	Estimate	*t*-value	*p*	*p*-adjusted
		High larvae density - Control	14.39	0.798	0.5333	0.9253
		Bti - Control	39.97	2.217	0.1137	0.2487
		**5 x Bti - Control**	62.29	3.455	0.0344	**0.0388**
		High larvae density - Low larvae density	13.23	0.734	0.5333	0.9435
		Bti - Low larvae density	38.81	2.152	0.1137	0.2715
		**5 x Bti – Low larvae density**	61.13	3.39	0.0344	**0.0429**
		Bti – High larvae density	25.58	1.419	0.3107	0.6306
		5 x Bti – High larvae density	47.9	2.656	0.0801	0.1321
		5 x Bti - Bti	22.32	1.238	0.3486	0.7314
Net CO_2 production (µmol d^{-1})	Treatment	Low larvae density - Control	109.6	2.095	0.1565	0.293
		High larvae density - Control	168.1	3.214	0.0464	0.0564
		Bti - Control	143.5	2.744	0.069	0.1159
		**5 x Bti - Control**	200.9	3.841	0.0213	**0.0326**
		High larvae density - Low larvae density	58.5	1.118	0.426	0.7938
		Bti - Low larvae density	33.9	0.648	0.6049	0.963
		5 x Bti – Low larvae density	91.3	1.746	0.2229	0.4512

Variables	Contrast	Pairwise structure	Estimate	*t*-value	*p*	*p*-adjusted
		Bti – High larvae density	-24.6	-0.47	0.6485	0.9885
		5 x Bti – High larvae density	32.8	0.627	0.6049	0.9671
		5 x Bti - Bti	57.4	1.097	0.426	0.8043
Ratio net CO_2 emission /net production		Low larvae density - Control	-0.1526	-1.374	0.2493	0.6558
		High larvae density - Control	0.0818	0.736	0.4786	0.9429
		**Bti - Control**	-0.4731	-4.26	0.0042	**0.0113**
		**5 x Bti - Control**	-0.5731	-5.16	0.0018	**0.003**
		High larvae density - Low larvae density	0.2344	2.11	0.0872	0.2872
		Bti - Low larvae density	-0.3205	-2.886	0.027	0.0934
		**5 x Bti – Low larvae density**	-0.4205	-3.786	0.0071	**0.0232**
		**Bti – High larvae density**	-0.5548	-4.996	0.0018	**0.0038**
		**5 x Bti – High larvae density**	-0.6548	-5.896	0.0015	**0.0011**
		5 x Bti - Bti	-0.1	-0.9	0.4323	0.8904
